# Protective effect of the olive hydroalcoholic extract on estrogen deficiency-induced bone loss in rats in comparison with estradiol 

**Published:** 2020

**Authors:** Farhad Koohpeyma, Mohammad Hossein Dabbaghmanesh, Mehrdokht Hajihoseini, Pedram Talezadeh, Nima Montazeri-Najafabady, Marzieh Bakhshayeshkaram

**Affiliations:** 1 *Shiraz Endocrinology and Metabolism Research Center, Nemazee Hospital, Shiraz University of Medical Sciences, Shiraz, Iran*; 2 *Health policy Research Center, Shiraz University of Medical Sciences, Shiraz, Iran*

**Keywords:** Black olive hydroalcoholic extract, Bone mineral density, Calcium, Phosphorus, ALP

## Abstract

**Objective:**

Osteoporosis, as a skeletal disorder caused by aging, is considered a major health problem. This work was planned to assess the effect of the black olive hydroalcoholic extract on bone mineral density and biochemical parameters in ovariectomized rats.

**Materials and Methods:**

Ninety 6-month-old female Sprague Dawley rats were randomly assigned into 7 sets: control (received saline); sham-operated control, Ovariectomized (OVX) rats (received saline); 3 groups of black olive-supplemented OVX rats (respectively, receiving 250, 500, and 750 mg/kg body wt black olive extract orally); and estrogen group (receiving 3 mg/kg/day estradiol valerate). Blood samples were collected 2, 4 and 6 months after treatment to measure calcium (Ca), alkaline phosphatase (ALP), and phosphorus (P). Dual-energy X-ray absorptiometry (DEXA) was applied to measure the bone mineral density (BMD). Global, lumbar spine and lower limb BMD was measured.

**Results:**

Ca concentration was significantly increased in the group treated with the highest dose of black olive hydroalcoholic compared to the OVX group (P<0.001). In addition, a significant decrease in ALP concentrations in the group treated with the highest dose of black olive hydroalcoholic comparing with the OVX group was observed (P<0.001). The global, tibia, femur and spine BMD in the group treated with the highest dose of black olive hydroalcoholic and estrogen group were significantly increased compared to the OVX group (P<0.05).

**Conclusion:**

Black olive hydroalcoholic extract at the dose of 750 mg/kg, prevented bone loss and augmented bone mineral density and could be a possible candidate for the management of osteoporosis.

## Introduction

Age-related diseases such as osteoporosis in developing societies cause morbidity (Tagliaferri et al., 2014 ▶). In bone tissues, both the formation and maintenance of the bone are regulated by bone-forming osteoblasts and bone-resorbing osteoclasts, where any imbalances lead to osteoporosis, a systemic skeletal disorder characterized by compromised bone strength and fracture (Bayat et al., 2018 ▶; Hamedani et al., 2015 ▶; Montazeri-Najafabady et al., 2018b ▶). There is rising evidence showing that in postmenopausal women, inflammation contributes to an early onset of osteoporosis. It was clearly defined that inflammatory cytokines such as Interleukin 1 (IL-1), IL-6, RANKL (Receptor activator of nuclear factor kappa-Β ligand), OPG (Osteoprotegerin), and M-CSF (macrophage colony-stimulating factor) are important elements in osteoclast differentiation and its bone resorptive activity (Ashouri et al., 2015 ▶). Now, most treatments aim to dramatically decline the bone resorption, leading to a higher net bone mineral density. Nonetheless, though effective, they have shown some side effects (Maraka and Kennel, 2015 ▶). Population studies have publicized that the incidence of osteoporosis in Europe is lower in the Mediterranean region (Benetou et al., 2013 ▶). The old Mediterranean diet, characterized by a high intake of fruits, vegetables and mostly olive oil, could be one of the ecological issues underlying this variance (Donini et al., 2015 ▶). In consideration of these studies, dietary intervention may suggest an advanced way to handle osteoporosis and its related complications. Olive oil is the main fat and one of the cornerstones of the Mediterranean diet. The favorable impacts of olive oil may be due to its components such as phenolic compounds, tocopherol, and carotenoids, which possess antimicrobial, antioxidant and anti-inflammatory properties (Cicerale et al., 2012 ▶). However, the main antioxidants of virgin olive oil are phenolic compounds including lipophilic and hydrophilic phenols (Covas, 2008 ▶). Phenolic compounds in the olive oil exert favorable impact on lipid oxidation, deoxyribonucleic acid (DNA) oxidative damage, and oxidative stress status (Cicerale et al., 2010 ▶).

Animal models of osteoporosis are suitable for assessment of a treatment’s efficacy and safety (Miller et al., 1995 ▶). Numerous therapeutic approaches for the management of osteoporosis were studied first in various animal models and then in clinical practice. The ovariectomized rat is the most widely used animal for reviewing the events linked with postmenopausal osteoporosis. Studies have shown that the rat skeleton is prone to the loss of ovarian hormones, make it a suitable for research (Lelovas et al., 2008 ▶). A significant and noticeable decreases in bone mineral density 10 weeks after OVX (Ovarectomized) was observed (Bauss and Dempster, 2007 ▶). BMD (Bone Mineral Density) has been expected to account for 60 to 70% of the bone strength variation (Howe et al., 2011 ▶). The combination of BMD and architectural variables can explain 90% of the variability in bone strength (Dempster, 2003 ▶). Previously, we reported the results of stereologic analysis of the impact of the black olive hydroalcoholic extract on osteoporosis in the vertebra and tibia in ovariectomized rats (Noorafshan et al., 2015 ▶). Here, we report the results of the analysis of the effects of the black olive hydroalcoholic extract on bone mineral density parameters to address another aspect of this issue.

## Materials and Methods

**Black olive hydroalcoholic extract**

Fresh black virgin olives which were randomly selected at optimum ripening time were obtained from Cultivation and Industry Company (Keshtvasanat, Fars, Iran). After seed removal, the olives were grounded to obtain a paste. Hydroalcoholic (50% ethanol and 50% distilled water v/v) extract of the 200 g black olive paste was prepared using the percolation method. The extracts were filter-sterilized using 0.45 μm membrane filters. Then to concentrate the extracts, the ethanol extracts were dried using rotary vacuum evaporator at 40°C (The efficiency of this method was 9%) (Noorafshan et al. 2015 ▶).

**Ovariectomy**

Bilateral ovariectomy and sham operation were done under anesthesia induced by ketamine 10% (100 mg/kg, Alfasan, Netherlands) and xylazine 2% (10 mg/kg, Alfasan, Netherlands). Then, the ovaries were excised after the ligation of uterine horn through a midline longitudinal incision in all the groups, except for the control group. The sham-operated control rats had their ventral incision, but manipulation of ovaries was performed without excising them.

**Animal care and treatment**

The tests performed in this study were carried out according to the rules in the Guide for the Care and Use of Laboratory Animals and was approved by the Ethics Committee of Shiraz University of Medical Sciences (No. 33-10358). One hundred five 6-month old female Sprague Dawley rats obtained from Shiraz University of Medical Sciences animal lab, weighing 200±20 g were used. The rats were fed with a standard diet, provided with water *ad libitum*, and maintained under standard housing laboratory conditions, relative humidity of 60±5%, temperature of 23±2°C, and 12 hr light/dark cycles. After a familiarization period of 1 week, they were randomly assigned into 7 groups (n=15 per group): group 1, control (received saline orally); group 2, sham-operated control (sham); group 3, OVX rats (received saline orally); and groups 4, 5, and 6 black olive-supplemented OVX rats (respectively, received 250, 500, and 750 mg/kg body wt black olive extract orally); group 7, estrogen [received 3 mg/kg/weekly estradiol valerate intravenously (Aburaihan pharmaceutical CO. Iran)]. Two months after ovariectomy, treatment was initiated and continued for 16 weeks (Noorafshan et al., 2015 ▶; Wronski et al., 1988 ▶; Wronski et al., 1989a ▶; Wronski et al., 1989b ▶).

**Biochemical measurement**

At the beginning and end of the experiment, blood samples collected into the chilled non-heparinized tubes, left for clotting and then centrifuged at 3500 rpm at 4°C for 20 min to prepare the serum. Calcium, phosphorus, and alkaline phosphatase (ALP), BUN and creatinine levels were measured using an enzymatic colorimetric method by a biochemical AutoAnalyzer device (mahmoodi et al., 2019 ▶). Kits were purchased from Pars Azmoon Co, Iran.

At the end of the study, the rats were sacrificed by terminal anesthesia by sodium thiopental intraperitoneally (100 mg/kg) and body weight and uterine wet weights were recorded.

**BMD measurements**

Dual-energy x-ray absorptiometry (DEXA) scans were done on a Discovery QDR, USA device, using specific software for small animals to evaluate the global, spine and lower limb BMD (g/cm^2^) at the first, three, and six months after treatment (Montazeri-Najafabady et al., 2018 ▶).

**Estimation of total phenolic content**

Total phenols were assayed according to Folin–Ciocalteu colorimetric method with minor modifications (Waterhouse, 2001 ▶). Appropriately, an aliquot of a 40 μl solution of the sample was added to 3.16 ml water and reacted with 200 μl Folin-Ciocalteu reagent for 8 min at room temperature. The reaction was then neutralized with sodium carbonate (600 μl of a 0.25% sodium carbonate) and allowed to stand for 2 hr in the dark at room temperature. Later, the absorbance of the resulting blue color was measured at 765 nm by a spectrophotometer. Quantification was done on the basis of a standard curve for gallic acid. The concentration of total phenolic compounds in the extract was expressed as milligram of gallic acid equivalent (GAE) per gram dry weight of extract, which was determined from a known concentration of gallic acid standard prepared similarly (Noorafshan et al., 2015 ▶). The total phenolic content in the 80% methanol extract was determined using the equation of the calibration curve and found to be 131.4±2.2 mg GAE/g dry extract (dE). 

**Statistical analysis**

All the statistical analyses were performed using the SPSS statistical software (v. 23). First, normality test was done. Then, the data were analyzed by Mann-Whitney U test. The results are presented as Mean±SD. Besides, differences were considered significant at p<0.05. 

## Results

**Biochemical parameters**

**Figure 1:**
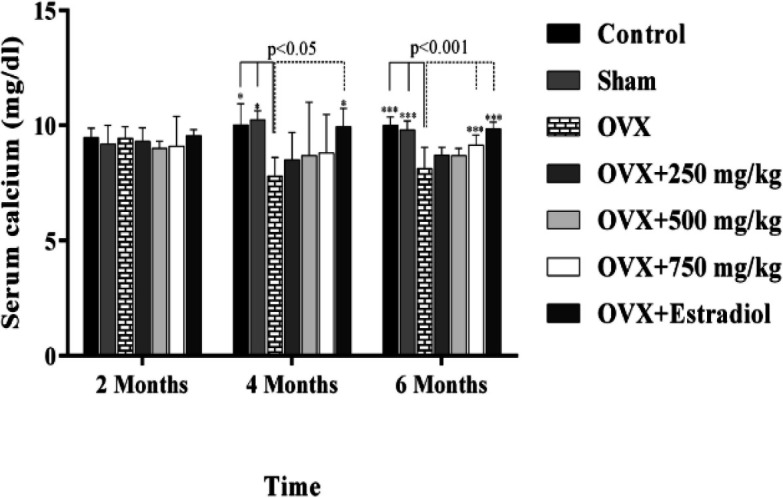
Effect of black olive hydroalcoholic extract and estradiol valerate on serum calcium (Ca) in ovariectomized rats after 2, 4 and 6 months of treatment. (*: P<0.05, ***: P<0.001)

Ovariectomy triggered a significant decrease in the calcium concentration in the untreated OVX group compared to the sham and controls in the final phases of the study (p=0.001). In addition, serum calcium concentration in the extract-treated and estrogen-treated animals were significantly greater compared to the OVX group 6 months after beginning of the experiment (P<0.001) (Figure 1). 

**Figure 2:**
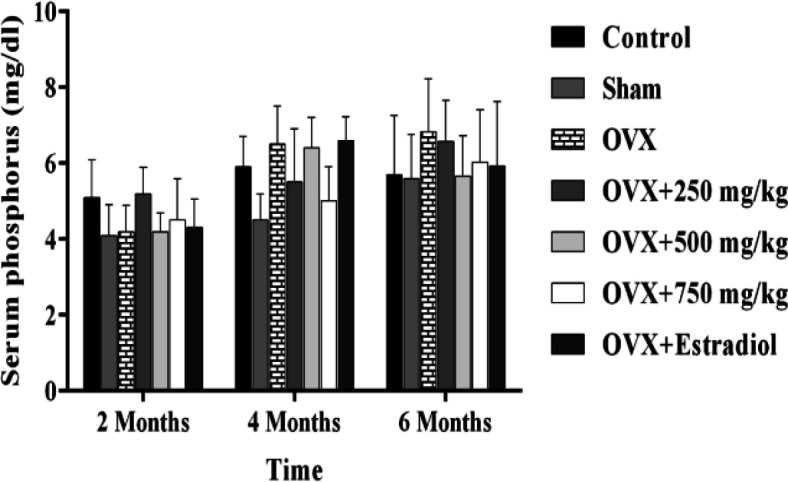
Effect of black olive hydroalcoholic extract and estradiol valerate on serum phosphorus (P) in ovariectomized rats after 2, 4 and 6 months of treatment

A significant difference in Ca concentration between estrogen-treated animals and OVX group was also observed 4 months after beginning of the experiment (P<0.05). Increasing the extract concentration enhanced the Ca concentration but there were no significant differences between various doses. Similarly, when we compared the serum phosphorus level among the control, OVX, extract-treated and estrogen treated animals in starting and final steps of the studies, no significant changes were found (Figure 2). 

**Figure 3:**
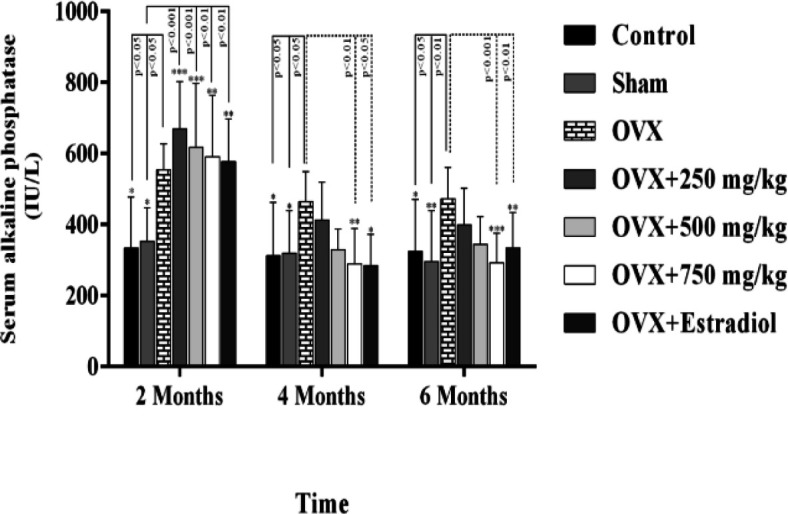
Effect of black olive hydroalcoholic extract and estradiol valerate on serum alkaline phosphatse (ALP) in ovariectomized rats after 2, 4 and 6 months of treatment

On the other hand, plasma ALP concentration significantly increased in the OVX rats compared to the sham-operated and control groups at the beginning of the study (p=0.001). After administration of black olive hydroalcoholic extract 750 mg/kg, there was a significant decrease in ALP level 2 and 4 months after beginning of the experiment (P<0.01). Also, there was a significant decrease in ALP concentration 2 (P<0.01) and 4 (P<0.05) months after beginning the experiment with estradiol (Figure 3). Increasing the extract concentration decreased the ALP concentration but there were no significant differences among various doses. No significant differences were observed between the black olive extract-treated groups and estrogen treated group in terms of Ca, P and ALP in this study at the final stage.

**Bone mineral density**

At the end of the experiment, global, spine and lower limb (tibia and femur) BMD were lower in the OVX group compared to the control and Sham groups, indicating the effectiveness of ovariectomy in our model. No significant differences were observed between the black olive extract-treated groups and estrogen-treated group at the final stage. 

**Global bone mineral density**

Global BMD was not significantly different between 250 mg/kg black olive extract-treated animals and the OVX group. However, the final global BMD was significantly lower in the OVX compare to the 500, and 750 mg/kg black olive extract-treated and estrogen-treated groups (P<0.05) (Table 1).

**Lower limb bone mineral density**

Lower limb showed significant differences between 250, 500, and 750 mg/kg black olive extract and estrogen-treated groups in the tibia BMD at the end of the experiment compared to the OVX group (P<0.05). Furthermore, a significant increase was observed for 500, and 750 mg/kg black olive extract and estrogen-treated groups in the femur BMD when compared to the OVX group at the end of the experiment (P<0.05). No significant differences in the tibia and femur BMD were observed between the OVX-treated with 250 mg/kg black olive extract and the OVX group, 2 months after beginning the experiment (Table 1).

**Table 1:** Effect of black olive hydroalcoholic extract and estradiol valerate on global, spine, femur, tibia BMD in ovariectomized rats 2, 4 and 6 months after beginning of the experiment

Parameter	periods of time (month)	Control	Sham	OVX	250 mg/kg	500 mg/kg	750 mg/kg	Estradiol
Glob-BMD g/cm2	2 4 6	0.165±0.008 0.177±0.006 0.173±0.006	0.164±0.008 0.174±0.004 0.173±0.005	0.165±0.004 0.168±0.005† 0.165±0.004†	0.162±0.006 0.167±0.005 0.168±0.003	0.163±0.005 0.171±0.007* 0.173±0.007*	0.166±0.003 0.175±0.008* 0.177±0.004*	0.165±0.004 0.178±0.005* 0.177±0.005*
Tibia-BMD g/cm2	2 4 6	0.092±0.013 0.090±0.012 0.093±0.024	0.126±0.160 0.090±0.012 0.111±0.013	0.068±0.019 0.067±0.016† 0.062±0.02†	0.079±0.018 0.082±0.01 0.96±0.025*	0.079±0.013 0.082±0.010* 0.111±0.013*	0.065±0.014 0.085±0.01* 0.109±0.024*	0.085±0.025 0.093±0.021* 0.113±0.02*
Femur-BMD g/cm2	2 4 6	0.174±0.007 0.188±0.019 0.178±0.010	0.172±0.014 0.179±0.014 0.178±0.015	0.167±0.013 0.166±0.015† 0.157±0.008†	0.164±0.01 0.173±0.009 0.165±0.035	0.169±0.015 0.184±0.015* 0.176±0.019*	0.170±0.009 0.191±0.012* 0.186±0.016*	0.171±0.11 0.184±0.007* 0.187±0.015*
Spine BMD g/cm2	2 4 6	0.197±0.011 0.206±0.008 0.202±0.012	0.200±0.016 0.206±0.011 0.204±0.011	0.202±0.011 0.190±0.012† 0.182±0.013†	0.193±0.01 0.196±0.011 0.194±0.011	0.193±0.012 0.199±0.014 0.195±0.019	0.202±0.015 0.207±0.008* 0.206±0.009*	0.197±0.011 0.214±0.011* 0.210±0.012*

†p< 0.05, OVX vs. (control or sham groups). *p<0.05, treated groups vs OVX. Data is presented as mean±SD.

**Spine bone mineral density**

Similarly, the spine BMD in the black olive extract-treated group at concentrations of 750 mg/kg and in the estrogen-treated group was significantly higher than that of the OVX group, 4 and 6 months after beginning of the experiment (P<0.05). No significant differences were observed in regard to spine BMD 2, 4 and 6 months after beginning of the experiment in the 250 and 500 mg/kg black olive extract-treated group compared to the OVX group (Table 1).

**Body and uterus weight**

**Figure 4:**
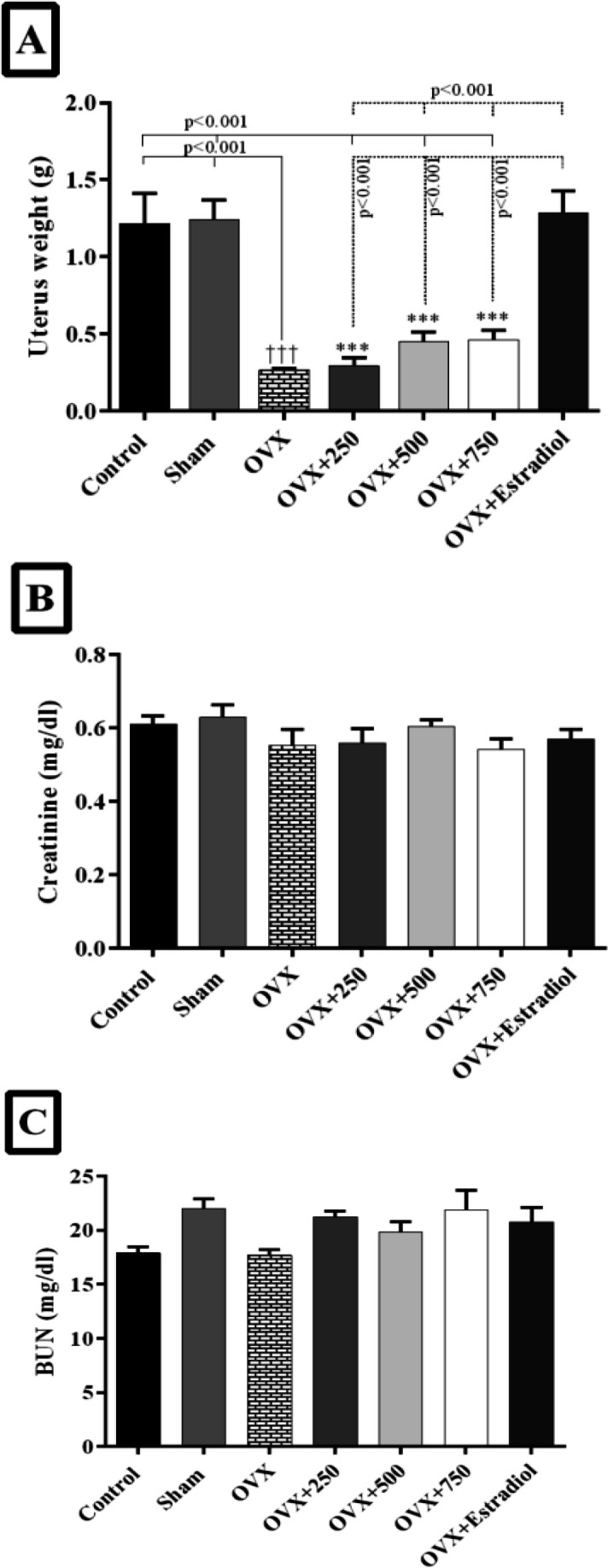
Effect of black olive hydroalcoholic extract and estradiol valerate on uterus weight, creatinine, and BUN in ovariectomized rats after 2, 4 and 6 months of treatment

The mean body weight in all rats at the beginning of the study was 200±20 g. After 6 months, a non-significant increase in body weight in the OVX group compared to the sham and control. Nonetheless, the weight of the uterus meaningfully decreased in the OVX rats compared to those with intact ovaries in the control and sham groups (p=0.001) (Figure 4).

**BUN and creatinine**

The serum BUN and creatinine concentrations in experimental groups are shown in Figure 4. No significant differences were observed between treated groups and OVX groups in terms of BUN and creatinine.

## Discussion

Postmenopausal osteoporosis is an age-related disease connected with low BMD and variations in bone microstructure (Dabbaghmanesh et al., 2017 ▶). A wide range of strategies has been designed to prevent and treat osteoporosis. Among them, nutrition and a healthy diet are the approaches that can be used because of their protective effect on the BMD (Maeda and Lazaretti-Castro, 2014 ▶). Olive and its components can prevent bone loss by different mechanisms in the experimental and *in vitro* models (Fernandez-Real et al., 2012 ▶). 

In the former study, we directed a stereological study on the effect of the black olive hydroalcoholic extract on osteoporosis in the vertebra and tibia in ovariectomized rats (Noorafshan et al., 2015 ▶).

In the present study, the effect of the black olive hydroalcoholic extract on biochemical factors was evaluated. Furthermore, we examined the effect of black olive hydroalcoholic extract on global BMD, spine BMD and lower limb BMD at three doses (250, 500, 750 mg/kg) in comparison with the estrogen-treated group.

In the current study, we found an increase in the body weight in all OVX rats compared to the control and sham groups; however, it was not significant. In line with our study, Kalu in 1991 showed that OVX rats had a higher weight in comparison with sham-operated rats (Kalu, 1991 ▶).

As reported previously, weight gain after oophorectomy is a protective mechanism for the skeletal system in case of estrogen deficiency (Zhao et al., 2013 ▶). It may be a possible explanation for the weight gain that we observed in our study.

Calcium homeostasis has a critical role in bone metabolism and calcium deficiency leads to impaired bone deposition (Saleh and Saleh, 2011 ▶). Our outcomes showed that calcium concentration was significantly elevated in the black olive hydroalcoholic extract and estrogen-treated group compared to the OVX rats. As previously reported, estrogen deficiency decreases the calcium absorption from the intestine and increases calcium excretion (Saleh and Saleh, 2011 ▶). It seems that black olive hydroalcoholic extract has a positive effect on ovariectomy-induced hypocalcemia. Increasing calcium concentration may be due to the effect of the black olive hydroalcoholic extract on enhancing the calcium absorption, and reducing calcium excretion and its impact on inhibiting the bone turn over markers (Saleh and Saleh, 2011 ▶), but further studies are needed to confirm this mechanism. 

Serum ALP, as the marker of bone remodeling, is increasing as a result of estrogen deficiency caused by ovariectomy (Noorafshan et al., 2015 ▶). In this study, the results showed that ALP was decreased after treatment with the black olive hydroalcoholic extract and estrogen compared to the OVX group. As we observed in our previous stereological study on the effect of black olive hydroalcoholic extract, regulating bone metabolism and decreasing the bone turn over makers, such as ALP, may be a possible mechanism underlying black olive hydroalcoholic extract effect (Noorafshan et al., 2015 ▶). In line with our results, Saleh and Saleh (2011) ▶ reported that ALP activity was decreased in the rats treated with olive oil (1 ml/12 g diet) compared to OVX (Saleh and Saleh, 2011 ▶).

As expected, similar to previous studies, we observed a lower global, spine, and lower limb (tibia and femur) BMD in the OVX group compared to the control and sham groups as a result of efficient ovariectomy. Consistent with our data, Kalu (1991) ▶ also reported the femoral bone loss in the OVX rats (Kalu, 1991 ▶). In addition, Cano et al. (2008) ▶ showed 4% femoral cortical BMD reduction after ovariectomy (Cano et al., 2008 ▶). Also, Paul et al. (2006) ▶ reported a reduction in the femoral BMD at the cancellous site. Estrogen deficiency that occurs after ovarian ablation leads to an increase in oxidative stress which finally results in bone loss (Puel et al., 2006 ▶). As reported previously, extra virgin olive oil and black Lucques olives, because of their phenolic components, neutralize and decrease oxidative stress prompted by estrogen deficiency. Black Lucques olives increased the total femur BMD in the OVX rats compared to the control group (Puel et al., 2007 ▶). Similarly, oral administration of hydroxytyrosol and oleuropein (antioxidant polyphenols) to mice protected them from ovariectomy-induced bone loss (Hagiwara et al., 2011 ▶). Saleh and Saleh found the protective effect of olive oil on ovariectomy-induced bone loss in Wistar rats. They also found that olive oil could decline serum IL-6, nitrate, ALP, and P (Saleh and Saleh, 2011 ▶). However, in our study, we did not detect significant differences between the black olive hydroalcoholic extract treated groups and OVX group in serum P.

In the current study, oral administration of black olive hydroalcoholic extract to the OVX rats, significantly increased global, tibia, femur, and spine BMD. Polyphenols that are present in olive hydroalcoholic extract regulate the bone metabolism by affecting osteoblasts and osteoclast. These compounds accelerated bone formation and mitigated bone resorption in OVX mice (Goto et al., 2015 ▶). Also, olive extract polyphenols have antioxidant potential to reduce the ovariectomy-induced oxidative stress which leads to an increase in reactive oxygen species (ROS) by decreasing inducible NO synthetase activity, an enzyme which is involved in producing NO (a ROS compound) (Liu et al., 2014 ▶). This gives a probable explanation for the significant increase in BMD observed in our study in the OVX rats treated with olive hydroalcoholic extract. In addition, it is well-defined that olive phenolic compounds, due to their anti-inflammatory properties, could prevent osteoporosis by modulating inflammatory variables (Puel et al., 2004 ▶). 

Interestingly, in another study, Keiler et al. (2014) ▶ observed that the total polyphenolic extract of extra virgin olive oil which was two times higher than normal concentration could not mitigate bone loss induced by estrogen deficiency (Keiler et al., 2014 ▶). In contrast, Tagliaferri et al. (2014) ▶ did not find significant differences in terms of BMD in the OVX mice treated with olive oil compared to the control group (Tagliaferri et al., 2014 ▶). Furthermore, the bone-sparing effect of either single olive polyphenols or olives in inflammation-induced models was reported (Puel et al., 2006 ▶; Puel et al., 2004 ▶).

Estrogen, as a sex steroid hormone, regulates the bone metabolism which is derived from its physiological function (Piri et al., 2016 ▶). Estrogen (especially estradiol) has a great impact on osteoblastic cell proliferation, DNA and protein content, and ALP activity (Garcia-Martinez et al., 2016 ▶) by binding to different estrogen receptors, including ERα and ERβ which results in expression of estrogen response genes (Zheng et al., 2016 ▶). 

Piri et al. showed a significant increase in BMD in the group of rats that received estrogen compared to the control group and they concluded that estradiol was directly associated with osteogenesis (Piri et al., 2016 ▶). Another study reported that daily subcutaneous administration of 17 β-estradiol at the lowest dose (10 μg/kg) could efficiently mitigate the bone loss in the OVX SD rats (Cano et al., 2008 ▶). In the current study, in the estrogen-treated group we found a significant increase in the global BMD, femur BMD, spine BMD compared to the OVX group after 4 and 6 months of treatment. In terms of the tibia BMD, a significant increase was observed only after 6 months. However, black olive extract-treated groups and estrogen-treated group showed significant increases in the BMD compared to the OVX group, but no significant difference was observed between the black olive extract-treated groups and estrogen-treated group in terms of BMD. Similarly, Zheng et al. indicated no significant difference between extra virgin olive oil group compared with the E_2_ (diethylstilbestrol) group (Zheng et al., 2016 ▶).

In this study, we did not find a significant difference between estrogen-treated group and OVX groups in term of phosphorus. It seems that estradiol at the levels used in this study was not effective in changing serum phosphorus. In another study, mice fed with olive oil had higher apparent calcium absorption and calcium balance, but a lower serum calcium, phosphate and magnesium level compared to groups fed with other lipids (Chin and Ima-Nirwana, 2016 ▶).

This study had some strengths and limitations. This study is the first study that evaluated the effect of black olive extract on BMD. As limitations, it was better to consider osteocalcin, bone morphogenic protein, N-telopeptide NTx, C-telopeptide CTx, deoxypyridinoline DPD pyridinium crosslinks, tartrate-resistant acid phosphatase TRAP, bone-specific alkaline phosphatase osteocalcin, P1NP procollagen type 1 N-terminal propeptide. Liu et al. compared the effectiveness of olive oil supplementation (1 ml/100 g diet) and diethylstilbestrol (25 ug/kg diet), a synthetic oestrogen mimicking oestrogen replacement therapy in humans, for 12 weeks in 6 months old OVX rats. Both treatments increased the BMD of lumbar spine and left femur of the ovariectomized rats. Similar to our results, they found that hypophosphatemia (probably due to ovariectomy-induced hyperparathyroidism), was not prevented by none of the treatments (Liu et al. 2014 ▶).

In conclusion, black olive hydroalcoholic extract at the highest concentration prevented bone loss and increased BMD of tibia, femur, and spine in this experimental model of osteoporosis. This effect was comparable to that of estradiol. 
